# How Live Streaming Features Impact Consumers’ Purchase Intention in the Context of Cross-Border E-Commerce? A Research Based on SOR Theory

**DOI:** 10.3389/fpsyg.2021.767876

**Published:** 2021-11-04

**Authors:** Jia Guo, Yu Li, Yujing Xu, Kai Zeng

**Affiliations:** School of Management, Zhejiang University of Technology, Hangzhou, China

**Keywords:** live streaming shopping, cross-border e-commerce, consumers’ purchase intention, saving money, SOR theory

## Abstract

Given that “cross-border e-commerce + live streaming” has become an important driver of global trade but limited attention has been paid to this area, this study examines the impacts of live streaming features on the consumers’ cross-border purchase intention from the perspectives of consumers’ overall perceived value and overall perceived uncertainty based on the SOR theory. In addition, through investigating the moderating effects of saving money, this study reveals the impacts of amazing bargains in live streaming commerce. A total of 272 samples were collected by a questionnaire survey to test the proposed research model. The results show that live streaming features significantly increase consumers’ overall perceived value and purchase intention, and significantly reduce consumers’ overall perceived uncertainty; in addition, saving money further increases the impact of live streaming features on consumers’ overall perceived value. This study provides a theoretical basis and reference for cross-border e-commerce platforms and merchants to effectively leverage live streaming to influence consumers’ perception and purchase intention.

## Introduction

According to the new released “Global Cross-Border B2C E-Commerce 2021” report (Research and Markets, 2021), cross-border online shopping is favored by a majority of consumers over the local online retail in 2020 because of its diverse products and attractive prices. Despite the COVID-19, the growth of cross-border e-commerce has maintained a relatively high speed. It is predicted that the overall cross-border e-commerce market value will surge by 30% from 2019 to 2026. For instant, in Italy, the majority of online shopping were performed across the borders, outweighing the domestic shopping in 2020. Due to the acceleration of cross-border e-commerce, it is necessary and important to understand consumers’ purchase intention and behavior in such context.

Existing research on cross-border e-commerce mainly focuses on traditional e-commerce elements (Martin et al., 2015; Agrawal and Fox, 2017; Zhang, 2018; Steinhoff et al., 2019). Limited attention has been paid to live streaming e-commerce. Live streaming, as a new diversified, real-time interactive medium, has been rapidly developed and widely used in cross-border e-commerce. Live streaming e-commerce refers to a marketing model in which streamers (sellers or their employees) rely on live streaming platforms to conduct online live streaming, and provide consumers with products descriptions and information through interpersonal communications and product trials, thereby promoting consumers’ purchase intention (Hu et al., 2017). Prior studies have found that the immediacy, interactivity and immersiveness of live streaming makes it more attractive to consumers than traditional online shopping modes (Liang et al., 2011; Haimson and Tang, 2017; Cai et al., 2018).

Despite the arising of both cross-border e-commerce and live streaming e-commerce, it is still unclear about the influencing mechanism of “cross-border e-commerce + live streaming” on the consumers’ purchase intention. Compared with traditional domestic e-commerce, “cross-border e-commerce + live streaming” has its own features. Firstly, given the complexity of cross-border transaction, the new “cross-border e-commerce + live streaming” mode enables consumers to fully understand the cross-border product and purchase process in real time, intuitively and in detail, which further improves consumers’ satisfaction. It is widely known that the transactions in cross-border e-commerce are more complex than domestic e-commerce. For instance, the transactional processes usually involve delivery risks and crossing language barriers, which may increase consumers’ perceived risks and uncertainties and deter consumers from taking full advantage of cross-border e-commerce (Koh et al., 2012; Guo et al., 2018). Luckily, live streaming could solve the above problem, which not only provides richer information to consumers through real-time responses and interactions with consumers, but also creates a hot selling atmosphere attracting consumers purchasing cross-border goods (Sun et al., 2019). Secondly, the streamers in the cross-border e-commerce context usually use price measures such as spikes and price reductions to stimulate and attract consumers. In the cross-border context, low price and good quality are the key factors that increase sales. According to a recent PayPal survey (2018), attractive prices are the reason why 72% of Australian consumers choose cross-border online shopping. Similarly, a report of Chinese cross-border e-commerce indicates that users pay more attention to genuine goods, service quality and low prices (iiMedia Research, 2021). Given above-mentioned unique natures of live streaming in cross-border e-commerce, it is quite meaningful to investigate (1) how the new “cross-border e-commerce + live streaming” mode affect consumers’ perceived uncertainty and perceived value, which further affect consumers’ cross-border online purchase intention and (2) what is the role of saving money in the relationships.

To address the research questions, this study examines the impacts of cross-border e-commerce live streaming features on the consumers’ purchase intention from the perspectives of consumers’ overall perceived value and overall perceived uncertainty based on the SOR theory. At the same time, through investigating the moderating effects of saving money, this study reveals the impacts of amazing bargains in the live streaming. A total of 272 samples were collected by a questionnaire survey, and then statistical analysis and hypothesis testing were carried out through structural equation models. The results show that live streaming features significantly increase consumers’ overall perceived value and purchase intention, and significantly reduce consumers’ overall perceived uncertainty. In addition, saving money further increases the impact of live streaming features on consumers’ overall perceived value. This study provides a theoretical basis and reference for cross-border e-commerce platforms and merchants to effectively leverage live streaming to influence consumer perception and purchase intention.

The findings of our study provide the following contributions to the existing literature. First, this study enriches the research by interpreting how live streaming can be used to affect consumers’ cross-border purchase intention based on SOR theory. In particular, we examined the influencing mechanism of “cross-border e-commerce + live streaming” on the consumers’ purchase intention from the perspectives of consumers’ overall perceived value and overall perceived uncertainty. Second, our study unveils the moderating effect of saving money on cross-border live streaming features affecting consumers’ overall perception. The results show that saving money can strengthen the positive impact of live streaming features on the overall perceived value while has no effect on the relationship between live streaming features and overall perceived uncertainty. Therefore, saving money is indeed a useful measure but should be used appropriately and carefully in live streaming. Our study also provides practical suggestions for cross-border e-commerce platforms and sellers to promote the continuous and healthy development of cross-border live streaming e-commerce.

## Theoretical Background and Hypothesis Development

### SOR Theory and Consumer Purchase

The SOR (Stimulus-Organism-Response) model was proposed by Mehrabian and Russell (1974) based on environmental psychology. The model believes that external factors would trigger a certain cognitive or emotional response and in turn lead to changes in consumer behavior (Jacoby, 2002). In recent years, with the vigorous development of live streaming e-commerce, scholars have explored the impact of live streaming on consumers’ purchase intentions and behaviors based on the SOR model. For example, Hu and Chaudhry (2020) used the SOR model to study how relational bonds can enhance consumer engagement.

**FIGURE 1:**
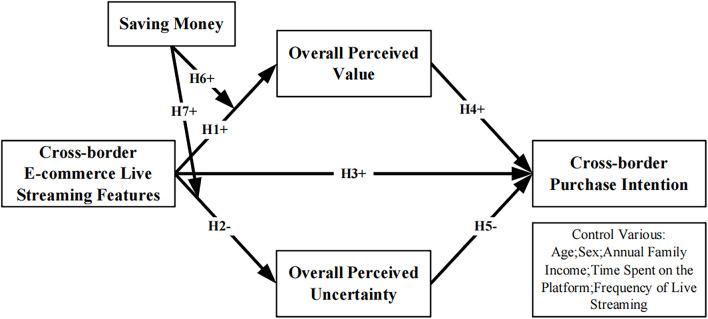
Research model.

Existing research mainly focuses on domestic live streaming e-commerce, and there has been a limited understanding of the impact mechanism of live streaming in the context of cross-border e-commerce. Therefore, based on the SOR theory, our study will use live streaming feature (S) as an external stimulus, and explore the mechanism of its influence on consumers’ cross-border purchase intentions from the perspectives of consumers’ overall perceived value and overall perceived uncertainty. In addition, considering the widely used promotion tools of saving money in the current live streaming scenarios, this article will also explore the moderating effect of saving money on cross-border live streaming features affecting consumers’ overall perception. The research model is shown in Figure 1.

### The Impacts of Live Streaming Features on Overall Perceived Value, Overall Perceived Uncertainty, and Purchase Intention

Live streaming features refers to the functions and features that live streaming could achieve real-time interaction through the comprehensive use of text, sound and image, and live streaming could deliver true and reliable information to consumers from multiple aspects, enabling consumers to clearly evaluate products’ performance (such as price, quality, and characteristics) (Zhou et al., 2018; de Wit et al., 2020). Cross-border live streaming e-commerce combines the advantages of multiple media, which not only enables sellers to transmit detailed and rich product information in real time (for example, production or procurement process, instructions for use), but also allows the streamers to communicate with consumers the feel, appearance, or smell of the products. The authenticity, visualization, and interactive performance displayed by the live streaming make consumers feel closer to cross-border products in space and time (Hu and Chaudhry, 2020).

According to the SOR theory, the live streaming features as an external stimulus will affect the cognitive response of consumers. Previous studies have pointed out that cognition can be divided into two dimensions: positive cognition and negative cognition in nature (Dubé and Menon, 2000). Guo (2020) also believes that when customers make purchase decisions, they not only care about prices, but also comprehensively compare the benefits and costs of available products. Therefore, this article will jointly understand the impact of live streaming features on consumers’ purchase intention from the two dimensions of consumers’ perceived value and perceived uncertainty in cross-border live streaming e-commerce.

Existing research points out that the structure of perceived value is multifaceted (Lee et al., 2002; Turel et al., 2007). Cocosila and Igonor (2015) believed that perceived value is related to the perception of product or service utility. Kim and Niehm (2009) proposed that perceived value includes a trade-off between the “obtained” part (the benefit that the buyer obtains from the seller’s product) and the “given” part (the cost paid by the buyer to purchase the offer). If people think that the gain exceeds what is given, they often feel satisfied. Chen and Lin (2018) believed that perceived value is a person’s feeling that certain objects or activities can bring benefits. Based on this, this study believes that the overall perceived value is constructed by multiple value perceptions, specifically referring to consumers’ overall judgment and evaluation of the transaction value and acquired value of the cross-border live streaming shopping according to their needs (Kim et al., 2012).

In addition, the perceived uncertainty in the network environment also plays an important role in the transactions between buyers and sellers (Yeh et al., 2012). Uncertainty refers to the degree to which the future environment cannot be accurately predicted due to imperfect information (Pfeffer and Salancik, 2015). As we all know, the perceived uncertainty in the e-commerce environment is high, especially in cross-border environments (Kim et al., 2017). This additional uncertainty may come from different factors, such as cross-border delivery (Guercini and Runfola, 2015), laws (Bieron and Ahmed, 2012), band (Guercini and Runfola, 2015) and online payment systems (Miao and Jayakar, 2016) and so on. In this study, the overall perceived uncertainty refers to the extent to which the buyer cannot accurately predict the outcome of the transaction due to factors related to the seller and/or cross-border products (Yeh et al., 2012).

Although research on domestic live streaming e-commerce is quite mature, the relationship between live streaming features and consumer perception in cross-border e-commerce still needs to be enriched. Cross-border live streaming e-commerce, as a main form of synchronized social media, has the advantages of high real-time, high interactivity and synchronization of communication (Wang and Wu, 2019), which not only provides useful information related to products or brands, but also delivers rich and interesting content, so the unique features of live streaming provide a more effective online shopping environment and stronger value perception than traditional media. Compared with domestic e-commerce, cross-border e-commerce platforms have higher information asymmetry and opportunism, which increase the overall perceived uncertainty of consumers (Yang and Shen, 2015). However, through live streaming, cross-border e-commerce demonstrates the use of products, displays different perspectives of products, and answers consumer questions in real time (Lu et al., 2018; Xu and Ye, 2020), having the characteristics of strong pertinence and low information loss, which can effectively reduce consumer perception of uncertainty. Based on this, this paper proposes the following research hypotheses:

H1: The live streaming features can enhance consumers’ overall perceived value in cross-border e-commerce platforms.
H2: The live streaming features can reduce consumers’ overall perception uncertainty in cross-border e-commerce platforms.

Purchase intention refers to the future intention and plan of customers to purchase the products or services they want (Aizen, 1991). Theory of reasoned action (TRA) believes that a person’s behavioral intention is affected by a person’s attitude toward the action and subjective judgments about the execution of the action (Chang, 1998). In this article, purchase intention refers to consumers’ intention to purchase cross-border products or services from sellers through live streaming (Sun et al., 2019).

The direct presentation of commercial information such as brands, products, prices, and promotional methods in cross-border live streaming e-commerce helps save consumers time for searching and comparison and improve their decision-making efficiency. At the same time, streamers rely on their professional skills to present cross-border products to consumers (Hu and Chaudhry, 2020), making consumers believe that the information in live streaming is more trustworthy than it in web pages of traditional e-commerce. The differentiated, professional, and personalized services provided by cross-border live streaming e-commerce help consumers fully understand the use and functional information of cross-border products and facilitate their purchase decisions. Based on this, this paper proposes the following research hypotheses:

H3: The live streaming features can enhance consumers’ purchasing intentions in cross-border e-commerce platforms.

### The Impacts of Overall Perceived Value and Overall Perceived Uncertainty on Purchase Intention

Regarding the relationship between overall perceived value and purchase intention, scholars pointed out that value perception factors such as service, entertainment, social interaction, finance, information, and image can directly affect purchase behavior (Chen and Lin, 2018; Hu and Chaudhry, 2020). Theory of Consumption Values believes that the motivation of consumers to participate depends on the value they expect to obtain from the experience. When customers think that they can obtain greater value from a product or activity, they will integrate more into the product or activity (Vivek et al., 2012). In contrast, due to the complexity of live streaming purchase decisions in cross-border e-commerce and the limited consumer perception, cross-border online shopping platforms, merchants, delivery companies, third-party payment and other external uncertainties will increase consumers’ worries, thereby reducing consumers’ willingness to buy (Yang and Shen, 2015). Based on this, this paper proposes the following research hypotheses:

H4: In the context of cross-border e-commerce, consumers’ overall perceived value has a positive impact on purchase intention.
H5: In the context of cross-border e-commerce, consumers’ overall perceived uncertainty has a negative impact on purchase intention.

### The Moderating Effects of Saving Money

Saving money refers to the extent to which consumers spend less or save money in cross-border purchases (Chiu et al., 2014). PayPal survey (2018) shows that price attraction is an important reason for consumers to choose cross-border online shopping. The cost of purchasing products or services has always been a general concern of consumers. In the traditional Chinese values and Confucian values, thrift is considered as a very important virtue (Cheung et al., 2003). However, in the context of cross-border e-commerce, whether giving consumers more concessions on prices helps to win more opportunities in the global market and how to win is worthy of further study.

Cross-border live streaming e-commerce can not only help consumers choose products more intuitively and conveniently, but also often launch various online promotion methods such as discounts, gifts and trials to increase consumers’ purchase possibilities. Wolfinbarger and Gilly (2001) pointed out that the pleasure of finding bargains is one of the reasons why individuals shop online. Therefore, the use of promotional methods to save consumers money can, to a certain extent, affect the impact of live streaming features on consumer perception in cross-border e-commerce. In the process of cross-border live streaming e-commerce, live streaming features can enable consumers to understand the products more clearly and enhance their perceived value. If cross-border live streaming e-commerce can save consumers money, it will further enhance consumer satisfaction and make consumers feel that they have gained more value. In addition, saving money is likely to further help live streaming features to reduce overall perceived uncertainty in cross-border e-commerce. In the process of cross-border live streaming, saving money can give consumers profit, and establish trust in cross-border live streaming and streamers, so that consumers can trust live streaming features and live streaming information, thereby effectively reducing their perceived uncertainty. Based on this, this paper proposes the following research hypotheses:

H6: In the context of cross-border e-commerce, saving money strengthens the positive impact of live streaming features on the overall perceived value.
H7: In the context of cross-border e-commerce, saving money strengthens the negative impact of live streaming features on the overall perceived uncertainty.

## Methodology

### The Samples and Data Collection

In order to test the research model and related hypotheses, this paper adopts a questionnaire survey method to collect data. It is noted that our study concentrates on consumers who have watched the live streaming of Alibaba’s Taobao Global Shopping and Tmall International e-commerce platforms. The reasons are as follows. First, Taobao Global Shopping and Tmall International Live streaming platforms play important part in Alibaba Group’s cross-border e-commerce platform, and their cross-border business in China and worldwide is very mature (Zhou et al., 2018). Second, Taobao Global Shopping and Tmall International e-commerce platforms are representative since they are frequently used and renowned to Chinese consumers.

Our study collects questionnaires through the Wenjuanxing app, which is the widely used data collection app in China. Since “cross-border e-commerce + live streaming” is a relatively new e-commerce field, we used a screening question to weed out respondents who have not been in contact with live streaming e-commerce or have not had any live streaming shopping experience. To guarantee the research quality, respondents who involved in the pre-survey are also excluded from the final survey. To express our gratitude, respondents who complete the survey will receive a reward of RMB 15 (equivalent to $2.14). In the end, we totally received 300 questionnaires. After removing questionnaires with too short answer time and failing the validity test, there are 272 valid questionnaires remaining.

### Measurements

In this paper, in order to ensure the accuracy and practicability of the questionnaire, all measurement items are based on the previous maturity scale and modified according to our research scenario. Among them, items of live streaming features refers to Zhou et al. (2018), items of overall perceived value refers to Kim et al. (2012), overall perceived uncertainty refers to Yeh et al. (2012), and items of money saving refers to Chiu et al. (2014), items of the purchase intention refers to Mou et al. (2019) and Sun et al. (2019). Likert’s seven-part scale is employed to measure each dimension, ranging from 1 (strongly disagree) to 7 (totally agree) to indicate the response to each construct related to the research questionnaire.

Since the survey was carried out for Chinese participants, this article conducts a forward-backward translation method to ensure the accuracy and consistency of the questionnaire presentation (Mullen, 1995). At the same time, in order to avoid the problem of common method bias (CMB), anonymous evaluation is used when designing the research program, and the length of the questionnaire is designed reasonably, while the order effect of the items is balanced. In addition, before the formal survey, this article conducted a preliminary survey of the questionnaire (*N* = 50) to explore the validity and readability of the questionnaire structure, and made appropriate revisions to the questionnaire based on the feedback to ensure the validity of the content of the scale.

## Data Analysis Results

### Descriptive Statistics

In the 272 valid survey samples, there are a total of 192 females and 82 males. More than 90.81% of the samples have a bachelor’s degree or above, 76.47% are people aged 20–29, and 77% of the samples have an annual household income of less than RMB 0.2 million. In the past 3 months, most people spent an average of less than 2 h on Tmall Global/Taobao Global e-commerce every days. The descriptive statistics of our survey samples are shown in Table 1.

**TABLE 1:** Descriptive statistics of the study sample.

Variable	Category	Frequency	Percentage (%)
Gender	Male	82	30.15
	Female	192	69.85
Age (years)	Less than 20	17	6.25
	20–24	140	51.47
	25–29	68	25
	30–34	20	7.35
	35–39	5	1.84
	40–44	9	3.31
	45–49	8	2.94
	More than 50	5	1.84
Education level	Secondary school or below	9	3.31
	Junior college	16	5.88
	Bachelor	172	63.24
	Master’s	71	26.10
	Ph.D.	4	1.47
Annual household income (RMB)	Less than 50,000	28	10.2
	50,000–100,000	68	25
	100,000–150,000	58	21.3
	150,000–200,000	56	20.5
	200,000–250,000	17	6.2
	250,000–300,000	18	6.6
	300,000–350,000	8	2.9
	350,000–400,000	6	2.2
	400,000–450,000	2	0.7
	450,000–500,000	3	1.1
	Above 500,000	8	2.9
Frequency of use (hours)	Less than 0.5	103	37.87
	0.5–1	96	35.29
	1–2	43	15.81
	2–3	18	6.62
	3–5	9	3.31
	Above 5	3	1.10

### Measurement Model Analysis

We use Cronbach’s alpha (CA) and composition reliability (CR) in the SmartPLS 3.0 software to test the consistency and stability of the scale. The results of the reliability test are shown in Table 2 (Note: Diagonal elements represent the square root of AVE). The CA values of all variables are between 0.798 and 0.931 and the CR values are between 0.882 and 0.956, all satisfying the standard of 0.7 (Fornell and Larcker, 1981), indicating that the research has good reliability.

**TABLE 2:** Cronbach’s alpha (CA), composite reliability (CR), average variance extracted (AVE), and correlations.

Constructs	CA	CR	AVE	LSF	OPV	OPU	SM	PI
Live streaming features (LSF)	0.872	0.913	0.725	0.851				
Overall perceived value (OPV)	0.869	0.911	0.718	0.356	0.847			
Overall perceived uncertainty (OPU)	0.931	0.956	0.879	−0.113	−0.102	0.938		
Saving money (SM)	0.858	0.914	0.779	0.219	0.516	0.149	0.883	
Purchase intention (PI)	0.798	0.882	0.714	0.472	0.515	−0.237	0.218	0.845

In this paper, the rationality and validity of the questionnaire are verified by structural validity test. The convergent validity is tested by using the average extracted variance value (AVE) and factor loadings. As shown in Table 2, the AVE values of all constructs are in the range of 0.714 to 0.879, which exceed the acceptable level of 0.5 (Fornell and Larcker, 1981), and the factor loadings of all constructs exceed the required value of 0.7, indicating that the data also satisfy convergent validity. Two methods are used to evaluate discriminant validity. The first method is the Fornell-Larcker criterion. Table 2 shows that the square roots of AVE exceed the correlation coefficient of each latent variable, confirming good discriminative validity (Fornell and Larcker, 1981). The second method is cross-loadings method. As shown in Table 3 (Note: The numbers in bold indicate the factor loadings of the construct items, and the numbers not in bold indicate the cross loadings of the construct items), all factor loadings exceed the cross-loading amount, again verifying the discriminant validity (Hair et al., 2018). As a result, the data shows a satisfied discriminative validity.

**TABLE 3:** Factor loadings and cross loadings.

Constructs	Items	Factor loadings and cross loadings				
		LSF	OPV	OPU	SM	PI
LSF	PLSF1	**0.788**	0.276	−0.111	0.228	0.299
	PLSF2	**0.896**	0.284	−0.074	0.153	0.447
	PLSF3	**0.920**	0.354	−0.105	0.213	0.481
	PLSF4	**0.793**	0.293	−0.101	0.157	0.351
OPV	OPV1	0.251	**0.836**	0.003	0.522	0.400
	OPV2	0.324	**0.871**	−0.064	0.447	0.427
	OPV3	0.285	**0.825**	−0.137	0.377	0.427
	OPV4	0.345	**0.857**	−0.151	0.399	0.491
OPU	OPU1	−0.097	−0.055	**0.935**	0.174	−0.226
	OPU2	−0.108	−0.147	**0.957**	0.114	−0.231
	OPU3	−0.114	−0.088	**0.921**	0.128	−0.210
SM	MS1	0.222	0.472	0.100	**0.871**	0.216
	MS2	0.241	0.445	0.114	**0.872**	0.234
	MS3	0.119	0.448	0.180	**0.905**	0.129
PI	ITP1	0.430	0.359	−0.244	0.138	**0.836**
	ITP2	0.470	0.477	−0.250	0.180	**0.907**
	ITP3	0.277	0.474	−0.091	0.244	**0.787**

*Factor loadings are in bold.*

Common method bias refers to the spurious variance between predictor variables and benchmark variables caused by the same data source, the same measurement environment, project context, and the characteristics of the project itself. Through reasonable design of our research process, the common method bias is controlled and reduced. At the same time, we also use the Harman’s single-factor method to identify common method bias. If the majority of the covariance can be explained by a single factor or a general factor, it is indicated that the common method bias is present. Principal component analysis extracted a total of 10 factors, of which the explanatory variance of the first principal component was 26.496%, which was lower than the standard of 40% (Chin et al., 2012), indicating that the data collected in this paper did not have obvious common method bias problems.

### Structural Model Analysis

**FIGURE 2:**
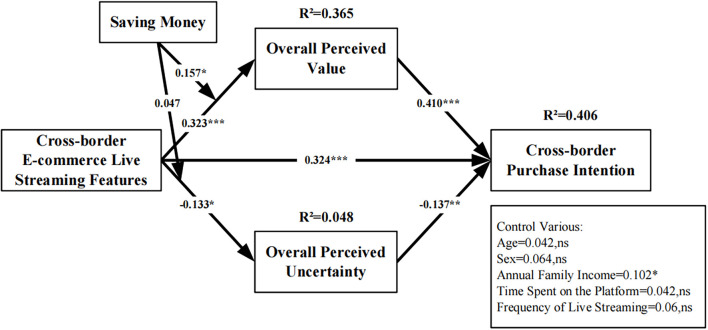
Results of the research model. *p < 0.05, **p < 0.01, and ***p < 0.001. n.s., not significant.

In order to obtain stable and reliable results, we used the bootstrapping algorithm of SmartPLS 3.0 to run 5,000 times to study the path coefficient and significance of each hypothesis. At the same time, the R^2^ of the related constructs was calculated, and the results are shown in Table 4 and Figure 2. The research results show that the R^2^ of overall perceived value, overall perceived uncertainty, and purchase intention are 36.5, 4.8, and 40.6%, respectively. The results show that live streaming features is positively associated with overall perceived value (β_1_ = 0.323, *P* < 0.001) and purchase intention (β_3_ = 0.324, *P* < 0.001) while negatively associated with overall perceived uncertainty (β_2_ = −0.133, *P* < 0.05) in cross-border e-commerce. That is, H1, H2, and H3 are supported. In addition, the results show that the overall perceived value (β_4_ = 0.410, *P* < 0.001) positively affects purchase intention in cross-border e-commerce, that is, H4 is supported. And the overall perceived uncertainty negatively affects purchase intention (β_5_ = −0.137, *P* < 0.01), that is, H5 is established.

**TABLE 4:** Main effects results.

Path	Path coefficient	Standard deviation	*T* value	*P* value	Hypothesis
LSF→OPV	0.323	0.062	5.198	0.000	H1 was supported.
LSF→OPU	−0.133	0.066	1.988	0.047	H2 was supported.
LSF→PI	0.324	0.060	5.350	0.000	H3 was supported.
OPV→PI	0.410	0.065	6.349	0.000	H4 was supported.
OPU→PI	−0.137	0.049	2.807	0.005	H5 was supported.

The results of the moderating effect are shown in Figure 2. The model uses age, gender, and annual household income as control variables to study the relationship between live streaming features, overall perceived value and overall perceived uncertainty, and purchase intentions of cross-border e-commerce. In addition, the impact of interactions between saving money and live streaming features on consumer perception is also studied (where LSF^∗^SMV refers to the moderating effect of saving money on the relationship between live streaming features and overall perceived value and LSF^∗^SMU refers to the moderating effect of saving money on the relationship between live streaming features and overall perceived uncertainty).

Figure 2 shows that, as a moderating variable, saving money strengthens the positive impact of live streaming features on the overall perceived value of consumers in cross-border e-commerce (β_6_ = 0.157, *P* < 0.05), that is, the H6 hypothesis is supported. However, contrary to our hypothesis, the moderating effect of saving money on the relationship between live streaming features and overall perceived uncertainty is not significant (β_7_ = 0.047, *P* > 0.05), that is, the H7 hypothesis does not hold.

## Conclusions and Implications

### Discussions and Conclusions

Based on the SOR model, this article explores how live streaming features affect consumers’ overall perceived value, overall perceived uncertainty, and purchase intention in cross-border e-commerce. We proposed 7 hypotheses, of which H7 is not supported, and the other hypotheses are consistent with the analysis results. In view of the empirical results, further discussion is as follows.

The findings that live streaming features can directly increase consumers’ purchase intention in the context of cross-border e-commerce are consistent with those of prior studies in the domestic e-commerce context (e.g., Zhang, 2018; Sun et al., 2019). In addition, the present study also finds that live streaming features have a positive impact on consumers’ overall perceived value while have a negative impact on consumers’ overall perceived uncertainty. That is, H1, H2, and H3 are established. According to the cumulative prospect theory (Tversky and Kahneman, 1992), consumers’ purchase intention is based on a full comparison of various decision-making factors. In the “cross-border e-commerce + live streaming” environment, consumers’ immersive shopping experience and the rich information provided by sellers are all subtly affecting consumers’ perception, which will increase the overall perceived value and reduce the overall perceived uncertainty of consumers. At the same time, the synchronization, visualization, and professional services provided by the live streaming will help consumers fully understand the use and functional information of cross-border products, and promote consumers to make purchase decisions.

Furthermore, the hypothesis test shows that the overall perceived value of consumers has a positive impact on purchase intention while the overall perceived uncertainty of consumers has a negative impact on purchase intention. That is, H4 and H5 are established. This is in line with the conclusions of Vivek et al. (2012) which pointed out that a higher level of perceived value will trigger purchase intention, and Yang and Shen (2015) which indicated that factors such as overall perceived uncertainty and purchase intention show a significant negative correlation. Whether it is a domestic e-commerce or cross-border e-commerce, the overall perception of consumers is an important indicator that affects purchase intentions. Sellers should pay more attention to consumers’ perception and consumers’ online shopping experience.

The research conclusion also shows that saving money can strengthen the positive impact of live streaming features on the overall perceived value, that is, H6 is established. This is consistent with the conclusion of Soscia et al. (2010) that saving money helps consumers obtain lower-cost products, thereby enhancing their value perception. However, the moderating effect of saving money on the relationship between live streaming features and overall perceived uncertainty is not significant, that is, H7 does not hold. Due to the complexity of cross-border e-commerce live streaming shopping (such as language issues, customs clearance issues, international delivery issues, etc.), consumers may associate negative words such as “low quality” and “unmarketable” with low prices when faced with cheap products or large discounts. Although saving money can give consumers profit, it will also make consumers question the quality of live streaming products, thereby increasing consumers’ perceived uncertainty. Those two effects of saving money canceled out leading to the insignificant moderating result of saving money on the relationship between live streaming features and overall perceived uncertainty. In summary, saving money is more like icing on the cake. A good cross-border e-commerce live streaming can enable consumers to understand the product more clearly and increase the perceived value of consumers. Under the circumstance, if measures of money-saving are implemented, it will further improve consumers’ satisfaction, thereby promoting consumers’ purchase behavior.

### Research Implications

The important implications of this research is to enrich the research of live streaming in the field of cross-border e-commerce and expand the application scope of SOR theory. The empirical results show that cross-border e-commerce platforms can indeed increase the overall perceived value of consumers through live streaming and reduce overall perceived uncertainty, thereby increasing consumers’ purchase intention. At the same time, the research also provides the following enlightenment for promoting the healthy development of “cross-border e-commerce + live streaming” mode.

First, this study verifies the important role of live streaming in cross-border e-commerce so that the practitioners should accelerate the construction of live streaming of cross-border e-commerce platforms, and actively explore optimization methods in live streaming to enhance consumer experience. Our study found that compared with traditional cross-border e-commerce operation models such as web pages or short videos, live streaming has features of higher authenticity and interactivity through the streamers’ description of the product’s feeling, appearance, or scent and other information. As a consequence, live streaming is able to increase consumers’ overall perceived value and reduce consumers’ overall perceived uncertainty, which in turn can increase consumers’ purchasing intentions. In addition, provided that live streaming is more mature in domestic e-commerce, the cross-border e-commerce market should draw on and learn from the successful experience of domestic live streaming e-commerce, accelerate the expansion of live streaming in cross-border e-commerce, and thereby improve and enrich consumers’ cross-border shopping experience. At the same time, the uniqueness of cross-border e-commerce live streaming should also be considered. Due to uncertain factors such as supply of goods, brand reputation, logistics and distribution capabilities in cross-border consumption, cross-border e-commerce platforms should strengthen the audit of cross-border products and improve the access threshold of live streaming products. Second, given that live streaming features can indeed increase consumers’ purchase intention in both direct and indirect ways, practitioners should make good use of the tool of live streaming. Our results show that live streaming features can effectively enhance consumers’ perceived value while reducing consumers’ perceived uncertainty. And those reminds cross-border e-commerce platforms and merchants that when embracing live streaming, they must strengthen the selection and professional training of cross-border e-commerce live streaming personnel, and pay attention to the output of live streaming content. More specifically, streamers should enrich the output content of live streaming and make high-quality content to attract consumers and eliminate consumers’ worries.

Third, our study would facilitate practitioners’ understanding regarding the effect of saving money in cross-border live streaming e-commerce. Our results indicate that saving money can indeed positively moderate the relationship between live streaming features and the overall perceived value of consumers in cross-border e-commerce, while the moderating effect on the relationship between live streaming features and the overall perceived uncertainty of consumers is not obvious. Therefore, saving money is more like icing on the cake and cross-border e-commerce platforms and streamers should take measures of saving money appropriately and carefully in live streaming. For example, cross-border e-commerce platforms and merchants should further strengthen their product quality control, so that consumers can trust low-priced products in cross-border live streaming e-commerce, and formulate reasonable money-saving strategies to stimulate consumers’ willingness to buy cross-border products.

### Limitations and Further Research

There are still some limitations in this study. First, the cross-border e-commerce platforms selected in this research are relatively mature cross-border e-commerce platforms, and future study could further expand and enrich the types of research subjects. Second, factors such as product types and streamers types can be introduced in future research to deeply explore and analyze the underlying mechanisms between live streaming and consumers’ perception and behavior. Last but not least, researchers can further explore the impacts of different degrees and methods of promotion tools in cross-border live streaming e-commerce.

## Data Availability Statement

The raw data supporting the conclusions of this article will be made available by the authors, without undue reservation.

## Ethics Statement

The studies involving human participants were reviewed and approved by the Secretariat of Academic Committee, Zhejiang University of Technology. The patients/participants provided their written informed consent to participate in this study.

## Author Contributions

JG, YL, YX, and KZ contributed conception and design of the study. JG and YX organized the data collection. YL performed the statistical analysis, wrote the first draft, and revised sections of the manuscript. JG, YX, and KZ polished the manuscript and contributed to the writing of manuscript revision. All authors contributed to the article and approved the submitted version.

## Conflict of Interest

The authors declare that the research was conducted in the absence of any commercial or financial relationships that could be construed as a potential conflict of interest.

## Publisher’s Note

All claims expressed in this article are solely those of the authors and do not necessarily represent those of their affiliated organizations, or those of the publisher, the editors and the reviewers. Any product that may be evaluated in this article, or claim that may be made by its manufacturer, is not guaranteed or endorsed by the publisher.
